# Retreatment with a pipeline embolization device for recanalized aneurysms following stent-assisted coiling embolization

**DOI:** 10.3389/fneur.2023.1267258

**Published:** 2023-11-03

**Authors:** Yuhui Ling, Jie Liu, Liyun Zhou, Xiuzhi Xiang, Peiming Wang

**Affiliations:** Department of Neurointervention, Guangdong Sanjiu Brain Hospital, Guangzhou, China

**Keywords:** pipeline embolization device, intracranial aneurysm, recanalization, stent-assisted coiling, antiplatelet therapy

## Abstract

**Background and purpose:**

Flow diverters have emerged as viable alternatives for the retreatment of recanalized aneurysms following stent-assisted coiling embolization. In this study, we aim to present our experience of retreatment for such aneurysms using the pipeline embolization device (PED).

**Materials and methods:**

This case series presents a retrospective single-center analysis of patients with recanalized aneurysms who underwent retreatment using the PED between July 2019 and April 2023, subsequent to stent-assisted coiling embolization.

**Results:**

The study includes five female patients, whose relevant clinical data were recorded. All patients had aneurysms located in the internal carotid artery, comprising two blood blister-like aneurysms and two giant aneurysms. Prior to the retreatment, two LVIS stents, two enterprise stents, and one solitaire stent were implanted. Among the five patients, one experienced a fatal post-operative subarachnoid hemorrhage, while two patients achieved complete embolization, and another patient achieved near-complete embolization during the last follow-up. Furthermore, one patient faced challenges during the placement of the PED and was unable to achieve successful deployment. We propose four overlapping relationships between a newly implanted PED and a previously deployed stent: (1) PED covering only the proximal end of the previous stent, (2) PED covering only the distal end of the previous stent, (3) PED covering both the proximal and distal ends of the previous stent, and (4) PED deployed within the previous stent. Antiplatelet therapy at our center involved daily dual therapy with aspirin (100 mg/day) and clopidogrel (75 mg/day) for at least 5 days before PED placement. Intra-arterial bolus administration of tirofiban (5 mcg/kg) was administered during or immediately after PED deployment, followed by a maintenance dose of 0.08 mcg/kg/min IV infusion for at least 24–48 h if necessary. Postprocedural dual antiplatelet therapy included clopidogrel (75 mg/day) for 6 months and aspirin (100 mg/day) for 12 months.

**Conclusion:**

The findings of this study support the efficacy of the PED for the retreatment of recanalized aneurysms following stent-assisted coiling embolization.

## Introduction

Advancements in endovascular techniques, such as coiling, stent-assisted coiling, and flow diverters (FD), have provided neurointerventionalists with viable alternatives to open surgery for managing complex aneurysms. Stent-assisted coiling has demonstrated relative safety and efficacy in reducing early recanalization rates and has shown superior effectiveness compared to coil embolization alone (1). Recanalization rates of stent-assisted coiling embolization for aneurysms range from ~8%−24% (1– 4). Nevertheless, treating recurrent-stented aneurysms remains a significant challenge for neurointerventionalists and neurosurgeons. A pipeline embolization device (PED) may serve as an alternative therapy for recurrent aneurysms after stent-assisted embolization. In the field of neurosurgery, the implementation of intraluminal stenting may result in heightened vessel rigidity and diminished maneuverability. On the other hand, procedures involving endovascular embolization, such as coiling and stent-assisted coiling, lack the capacity to rectify hemodynamic issues. Therefore, it would be ill-advised to undertake repeated unsuccessful interventions for the same aneurysm.

However, the safety and effectiveness of PED treatment for recurrent aneurysms after stent-assisted embolization have not been conclusively established (5–9).

This study presents five representative cases from our center, comprising two giant aneurysms and two blood blister-like aneurysms, all located on the posterior communicating artery (P-comm) region. Patient 3 initially received treatment at our center, while the remaining patients had initial stent-assisted coiling procedures at other hospitals. All PEDs implanted in this case series were second-generation devices—pipeline flex (Medtronic, Minneapolis, Minnesota).

## Materials and methods

The study received approval from the institutional research ethics boards of Guangdong Sanjiu Brain Hospital. We conducted a review of medical records and image data from our aneurysm database, which comprised patients diagnosed with intracranial aneurysms between January 2017 and June 2023. All patients included in this study met the following inclusion criteria: (1) intracranial aneurysms confirmed by digital subtraction angiography (DSA) and treated with endovascular procedures, (2) recanalization observed during follow-up angiography and subsequently retreated with endovascular treatment, and (3) further follow-up angiographic imaging to determine whether the aneurysm had recanalized after retreatment. The recorded data encompassed patient demographics (age and gender), presenting symptoms, clinical status, aneurysm characteristics, endovascular details, treatment-related complications, and neurological outcomes as assessed by the modified Rankin Scale scores at discharge and during the last follow-up.

The management and planning of endovascular treatment for all patients in this series were based on a multidisciplinary neurosurgical review of the patient's neurovascular and pathologic anatomy. During the peri-operative and post-operative periods, all patients were administered aspirin and clopidogrel. Systemic heparinization was employed during the endovascular procedures.

## Results

**Figure 1:**
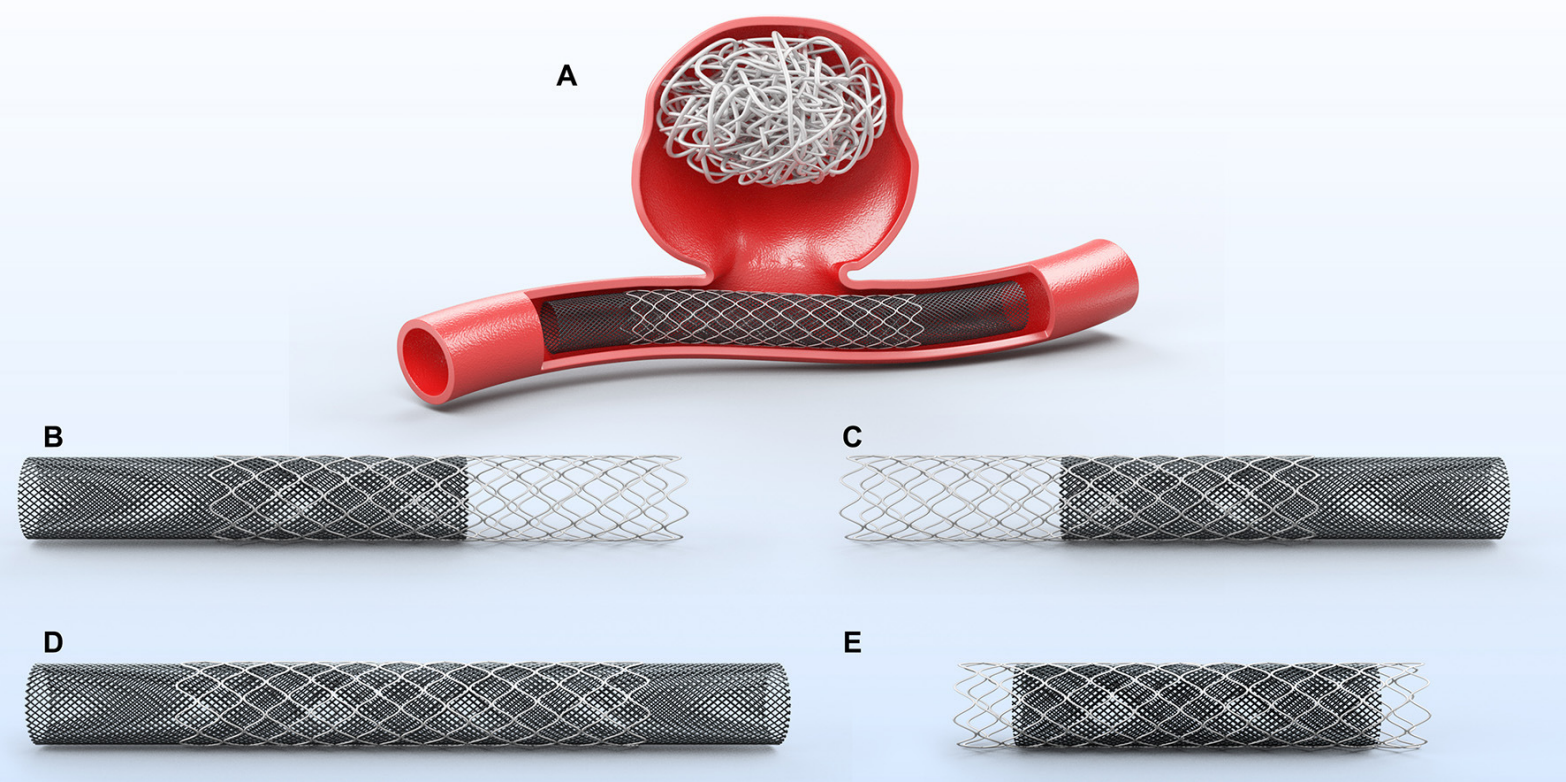
Illustration of retreatment. **(A)** Retreatment with pipeline embolization device (PED) for recanalized aneurysm after stent-assisted coiling embolization. Four types of overlap between a newly implanted PED and a previously deployed stent. **(B)** PED only covers the proximal end of the previous stent. **(C)** PED only covers the distal end of the previous stent. **(D)** PED covers both the proximal and distal ends of the previous stent. **(E)** PED is deployed within the previous stent.

The study includes five female patients, whose relevant clinical data were recorded. All patients had aneurysms located in the posterior communicating segment of the internal carotid artery, comprising two blood blister-like aneurysms and two giant aneurysms. Prior to the retreatment, two LVIS stents, one enterprise stent, and one solitaire stent were implanted. Among the five patients, one case experienced a fatal post-operative subarachnoid hemorrhage, while two cases achieved complete embolization, and one case achieved near complete embolization at the last follow-up. Relevant information is shown in Table 1. Illustration of retreatment and four types of overlap between a newly implanted PED and a previously deployed stent are shown in Figure 1.

**Table 1:** Patient data and aneurysm characteristics.

**Case no**.	**Sex**	**Age (years)**	**SAH**	**Aneurysm size (mm)**	**Aneurysm location**	**Blood blister-like**	**Prior stent**	**Time from initial treatment (months)**
Patient 1	Female	49	Yes	6.5^*^9.3	Left ICA	Yes	LVIS^*^1	6
Patient 2	Female	48	Yes	45.2^*^43.7	Left ICA	No	Enterprise^*^1	7
Patient 3	Female	35	Yes	2.0^*^2.5	Right ICA	Yes	LVIS^*^1	13
Patient 4	Female	56	Yes	24.5^*^25.8	Right ICA	No	Solitaire^*^1	11
Patient 5	Female	55	Yes	16.0^*^13.2	Right ICA	No	Enterprise^*^1	21

No., number; SAH, subarachnoid hemorrhage (initial treatment); P-comm, posterior communicating artery. ^*^ means that the use of one stent.

### Patient #1

**Figure 2:**
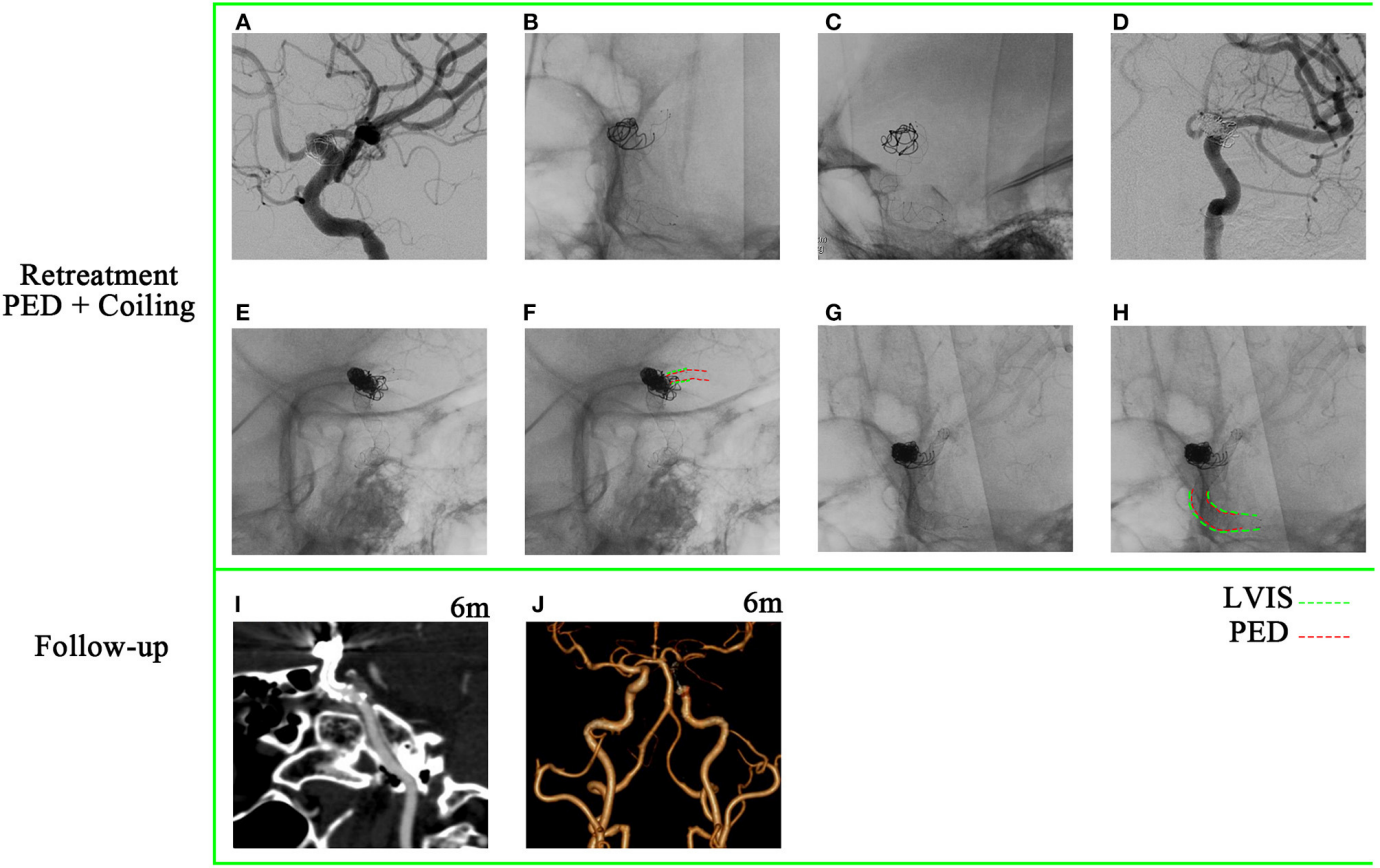
A 49-year-old woman with a recanalized blood blister-like aneurysm. **(A)** Digital subtraction angiography revealed a recurrent posterior communicating artery aneurysm with a maximum diameter of 9.3 mm. **(B**, **C)** Previously placed LVIS stent and loose coils. **(D)** Aneurysm achieved near-complete occlusion through pipeline embolization device-assisted coiling. **(E**–**H)** Pipeline embolization device covered only the proximal end of the previous LVIS stent. **(I, J)** A 6-month follow-up computed tomography angiography (CTA) indicated complete occlusion.

A 49-year-old woman underwent stent-assisted coiling (LVIS) for a P-comm blood blister-like aneurysm at another hospital 5 months ago. She was admitted to our hospital for treatment of recurrent aneurysm, which was diagnosed during a computed tomography angiography (CTA) scan performed 2 weeks ago. DSA revealed a recurrent aneurysm with a maximum diameter of ~9.3 mm (Figure 2A). The previous LVIS stent and loose coils are shown in Figures 2B, C, respectively. The aneurysm was retreated using a PED of 4.0 mm × 20 mm (Medtronic, Minneapolis, Minnesota) and three coils. A complete occlusion of the recanalized aneurysm was achieved (Figure 2D). The PED covered only the proximal end of the LVIS stent (Figures 2E, F). A 6-month follow-up CTA indicated complete occlusion.

### Patient #2

**Figure 3:**
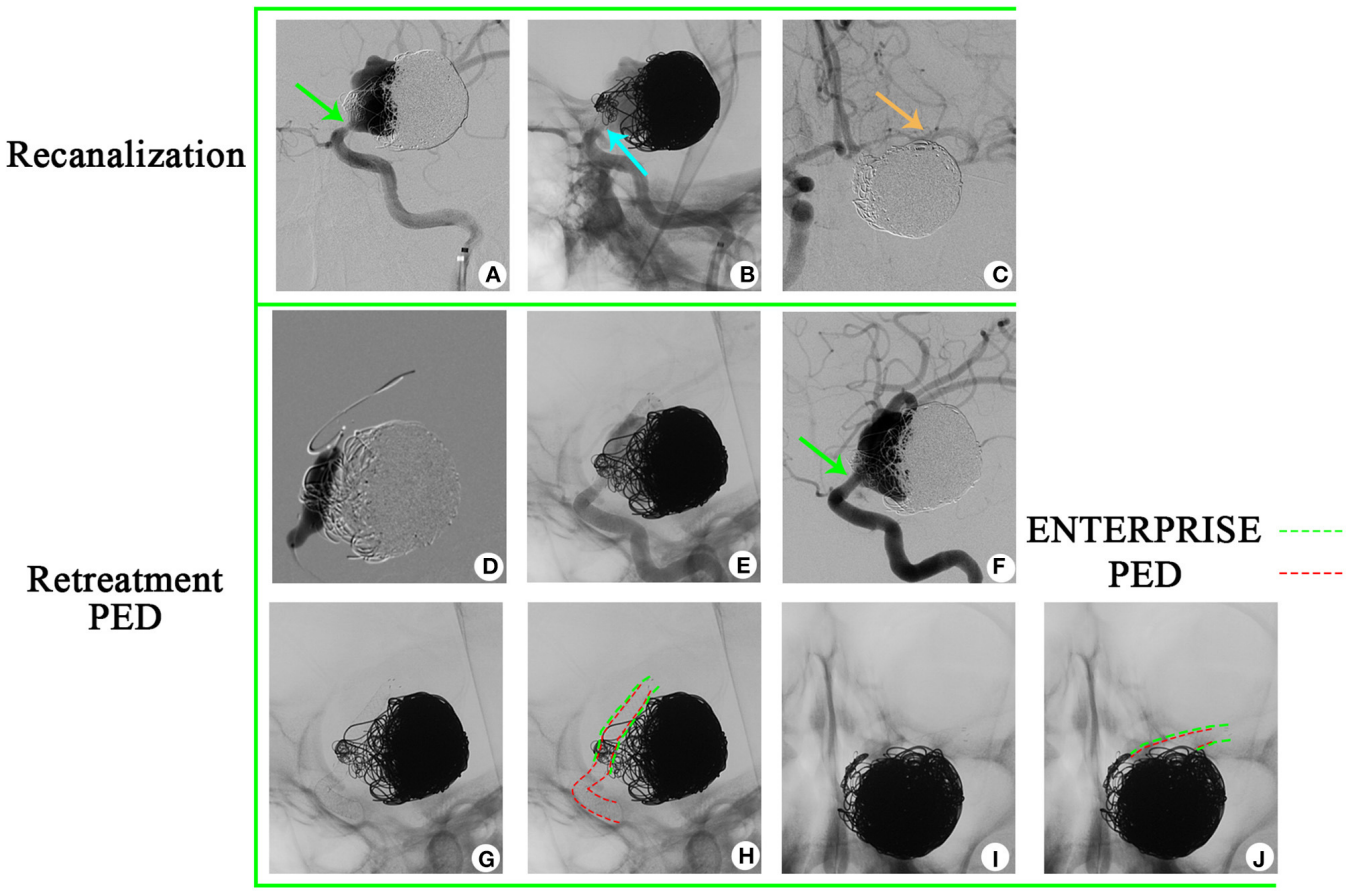
A 48-year-old woman has been diagnosed with a recurrent giant aneurysm. **(A–C)** The aneurysm is located in the posterior communicating artery, with a size of 45.2 × 43.7 mm, as observed in digital subtraction angiography. **(A)** 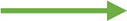 Stenosis of the proximal parent artery was indicated. **(B)** 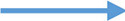 Images showed that the proximal markers of the enterprise were visible in the neck of the aneurysm. **(C)** 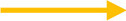 Aneurysm is not involved in the bifurcation of the internal carotid artery. **(D)** Balloon angioplasty was performed to address the stenosis of the proximal parent artery. **(E)** A pipeline embolization device was deployed. **(F)** Relief of the stenosis. **(G–J)** The pipeline embolization device was observed to cover only the distal end of the enterprise.

A 48-year-old woman presented with a sudden acute onset of headache, nausea, and vomiting. Emergency CT revealed subarachnoid hemorrhage, and CTA showed a ruptured P-comm aneurysm. She achieved aneurysm occlusion through stent-assisted coiling (Enterprise) at another hospital and was discharged without any neurological deficit. A 9-month follow-up DSA showed a major recurrence (Figures 3A–C). The aneurysm size was 45.2 × 43.7 mm. The stenosis of the proximal parent artery was confirmed (Figure 3A). Proximal markers of the Enterprise were seen in the aneurysmal neck (Figure 3B), and the aneurysm was not involved in the bifurcation of the ICA (Figure 3C). Retreatment of the giant aneurysm was performed with a PED (Medtronic, Minneapolis, Minnesota) of 4.5 mm × 35 mm after balloon angioplasty (Figures 3D–F). The stenosis of the proximal parent artery was relieved (Figure 3F). The PED covered only the distal end of the enterprise stent (Figures 3G–J). However, the patient suddenly experienced a severe headache and loss of consciousness 1 h after the operation. CT showed SAH, and the patient passed away 2 days later.

### Patient #3

**Figure 4:**
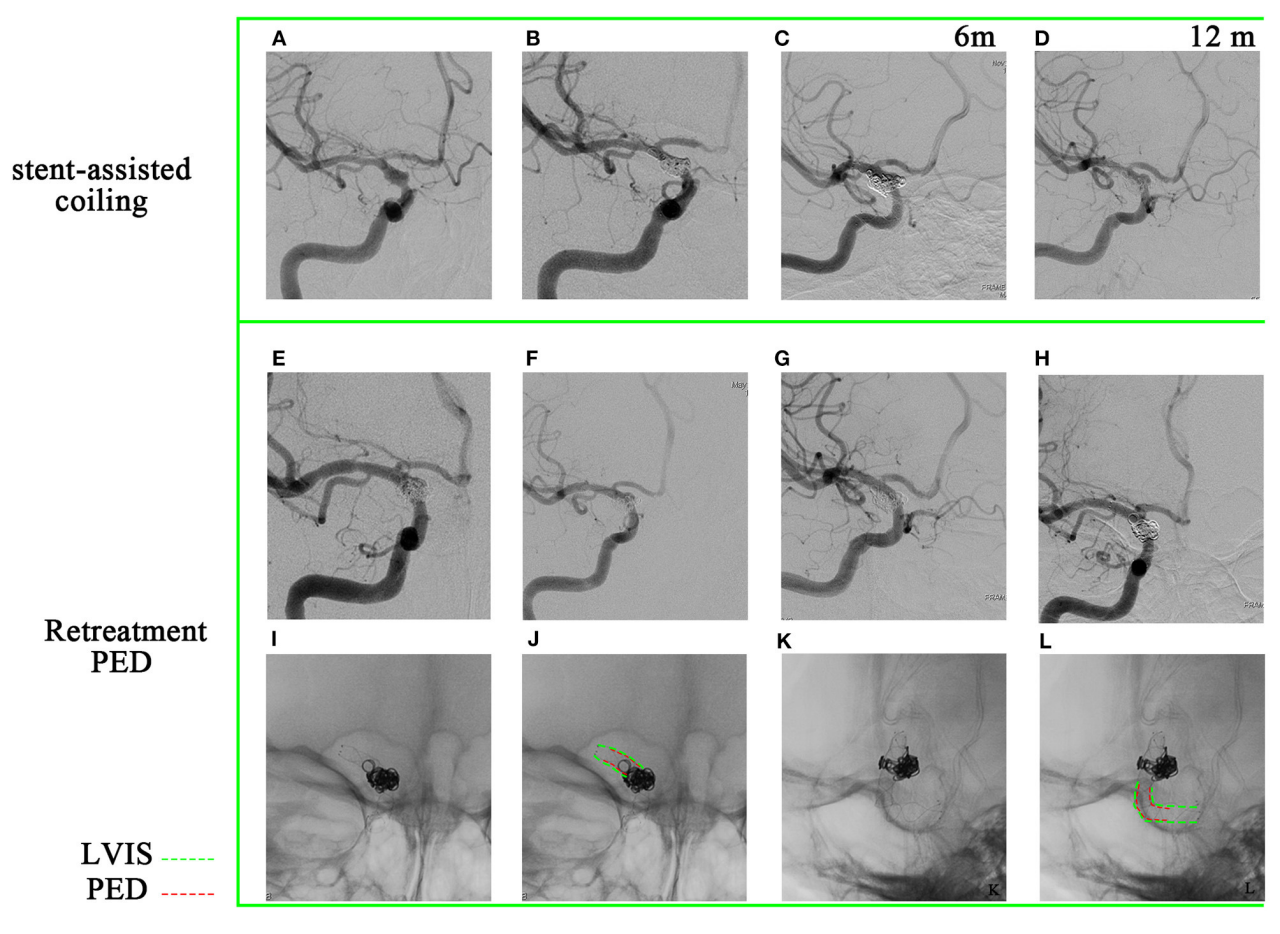
A 36-year-old woman has been diagnosed with a recurrent blood blister-like aneurysm. **(A)** The aneurysm is located in the posterior communicating artery and has dimensions of 2.0 × 2.5 mm, as observed in digital subtraction angiography. **(B)** To treat the aneurysm, a stent-assisted coiling (LVIS) procedure was performed, achieving near-complete occlusion of the aneurysm. **(C, D)** A 6-month and 12-month follow-up angiography confirmed that the aneurysm had recanalized. **(E, F)** Deployment of a pipeline embolization device. **(G, H)** The 12-month follow-up after retreatment angiography demonstrated complete occlusion of the aneurysm. **(I–L)** The PED is a pipeline embolization device within the previous LVIS.

A 36-year-old woman presented with a sudden acute onset of headache, nausea, and vomiting. Emergency CT revealed subarachnoid hemorrhage, and CTA showed a ruptured P-comm aneurysm. DSA demonstrated a BBA of P-comm, with a size of 2.0 × 2.5 × 3.0 mm (Figure 4A). Stent-assisted coiling was performed (LVIS; MicroVention, 4.0 × 20 mm; Figure 4B), and four coils were used. A 6-month follow-up angiography after LVIS stent implantation confirmed recanalization of the aneurysm (Figure 4C), and a 12-month follow-up angiography revealed aggravation of recanalization (Figure 4D). The BBA was then retreated with a PED (Medtronic, Minneapolis, Minnesota, 3.0 mm × 16 mm; Figures 4E, F). A 12-month follow-up after retreatment showed complete occlusion (Figures 4G, H). The PED was implanted within the LVIS stent (Figures 4I–L).

### Patient #4

**Figure 5:**
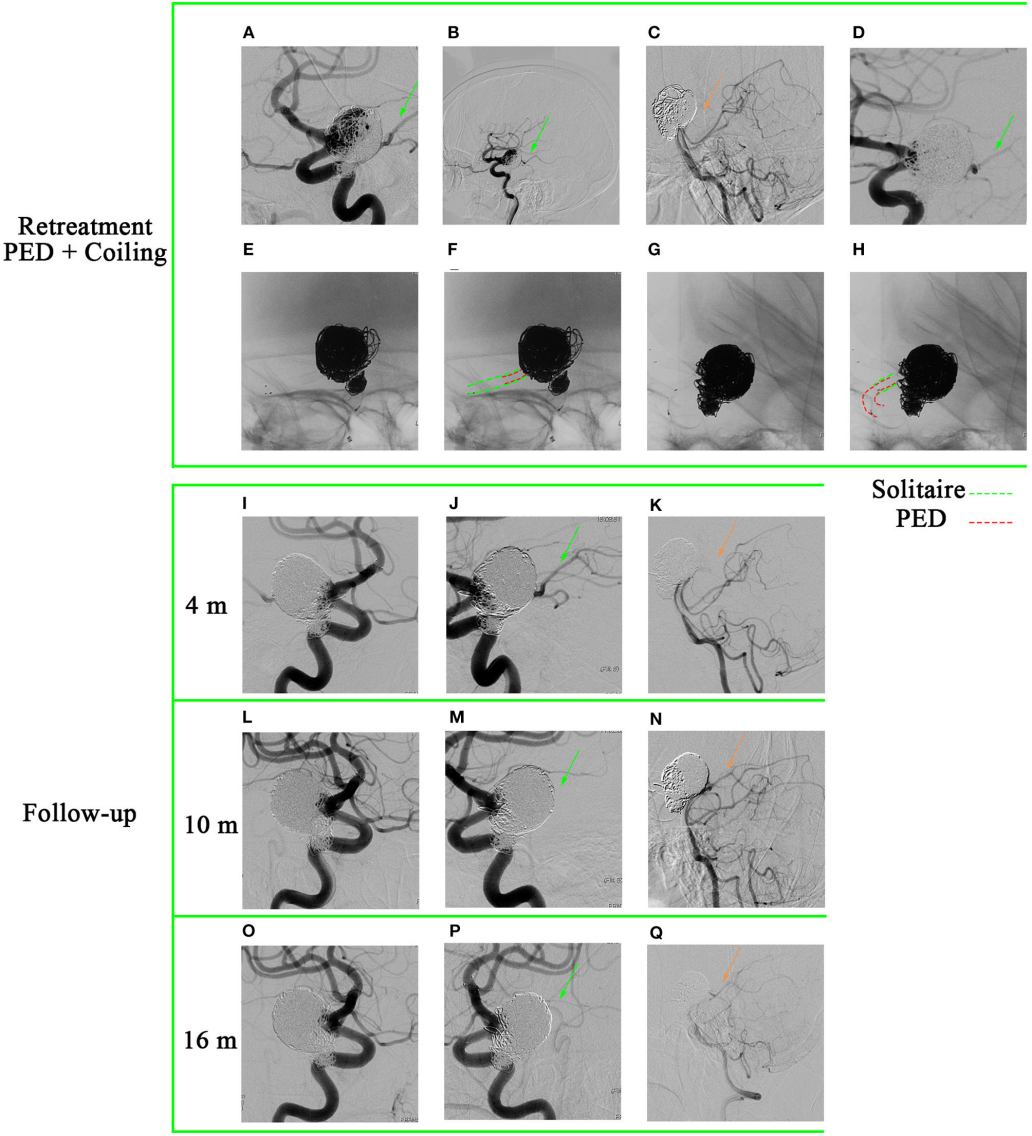
A 56-year-old woman has a recurrent giant aneurysm, specifically in the posterior communicating artery. **(A–C)** Digital subtraction angiography revealed the size of the aneurysm to be 24.3 × 25.8 mm. It has been confirmed that the patient has bilateral patent right fetal PCAs (posterior cerebral arteries). 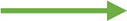 indicates the presence of right PCA under injection of the right internal common artery. 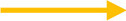 indicates the absence of right PCA under injection of the right vertebral artery. **(D)** Implantation of pipeline embolization device. 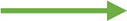 indicates the presence of right PCA under injection of the right internal common artery. **(E–H)** The pipeline embolization device covers the proximal end of the previous Solitaire device. **(I–Q)** The -month, 10-month, and 16-month follow-up angiographies are shown. **(J, M, P)** Gradual occlusion of the right posterior communicating artery. **(K, N, Q)** Gradual opening of P1 segment of right PCA **(K, N, Q)**. 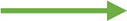 indicates right PCA under injection of the right internal common artery; 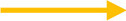 indicates P1 segment of right PCA under injection of the right vertebral artery.

A 56-year-old woman presented with a sudden acute onset of headache. Emergency CT showed subarachnoid hemorrhage, and CTA revealed a ruptured right P-comm aneurysm. She received stent-assisted coiling (Solitaire) for the aneurysm at another hospital and was discharged without any neurological deficit. A 3-month follow-up DSA indicated a major recurrence (Figures 5A–C). Bilateral fetal PCAs were confirmed, and the right PCA was shown in Figures 5B, C. Retreatment of the giant aneurysm was performed with a PED (Medtronic, Minneapolis, Minnesota) of 4.5 mm × 35 mm and six coils after balloon angioplasty (Figure 5D). The PED covered only the proximal end of the solitaire device (Figures 5E–H). A 4-month (Figures 5I–K), 10-month (Figures 5L–N), and 16-month (Figures 5O–Q) follow-up angiographies after PED implantation were shown. Injection into the right internal carotid artery demonstrated: (1) gradual healing of the recanalized aneurysm and (2) occlusion of the right P-comm (Figures 5M, P). Injection into the right vert artery demonstrated the reopening of the P1 segment of the right posterior cerebral artery (Figures 5N, Q). During the patient's retreatment process, gradual occlusion of the posterior communicating artery was accompanied by a gradual opening of the P1 segment of the posterior cerebral artery. No new neurological symptoms were observed throughout the process, demonstrating the advantages of flow diversion devices in treating aneurysms. This approach slowly occludes the aneurysm while allowing time for compensatory collateral circulation to develop, thus minimizing the risk of ischemia. A 4th-month follow-up DSA of patient #4 showed that the residual contrast agent in the tumor neck was more obvious than immediately after the PED implantation (Figures 5D, I). Consequently, we decided to stop clopidogrel and only use aspirin. A 10-month follow-up DSA after PED implantation showed that the aneurysm neck gradually healed (Figure 5L). After stopping aspirin, DSA showed further healing of the aneurysm neck at 16 months (Figure 5O).

Radiographic and clinical outcomes following PED deployment are shown in Table 2.

**Table 2:** Radiographic and clinical outcomes following PED treatment for recurrent previously stented aneurysms.

**Case no**.	**mRS**	**Angiographic follow-up (months)**	**Complication**	**Occlusion on latest follow-up**	**Stenosis of parent artery**
Patient 1	0	6	None	Complete	No
Patient 2	6	–	SAH	–	–
Patient 3	0	12	None	Complete	No
Patient 4	0	16	Occlusion of branch vessel	Near complete	No
Patient 5	0	–	–	–	–

No., number; mRS, modified Rankin score; SAH, subarachnoid hemorrhage; PED, pipeline embolization device.

## Discussion

In this case series, we present five cases of retreatment using the PED for recanalized aneurysms of the posterior communicating artery after stent-assisted coiling embolization.

The PED was initially approved by the Food and Drug Administration (FDA) for use in adults with giant and large aneurysms extending from the petrous to superior hypophyseal segments of the internal carotid artery in April 2011. However, off-label uses of PED in refractory and complicated aneurysms, such as large or giant, dissecting, blood blister-like, and recanalized aneurysms, have gained increasing acceptance (10). The safety and efficacy of PED implantation in a pre-existing stent remain unclear as data from different studies vary greatly. While previous studies indicated high recanalization rates with stent-assisted coiling, ranging from ~57% to 78% (7, 11), Park et al. (12) reported a 94% complete closure rate of PED for recurrent aneurysms after stent-assisted coiling. Given the complexity of recurrent aneurysms after stent-assisted coiling, these data suggest that PED may be the optimal alternative.

PED implantation for recurrent aneurysms after stent-assisted coiling embolization is technically challenging and sometimes dangerous. Some studies reported procedural events related to implanting PED in a previous stent, with no corresponding scheme provided to avoid these technical difficulties (6, 13). The strategy to deploy the PED remains controversial (5, 13, 14). There are four types of overlap between a newly implanted PED and a previously deployed stent: (1) PED only covers the proximal end of the previous stent, (2) PED only covers the distal end of the previous stent, (3) PED covers both the proximal and distal ends of the previous stent, and (4) PED is deployed within the previous stent. Different researchers propose different approaches. Chalouhi et al. (5) deployed the PED within the indwelling stent or ensured sufficient PED length to anchor on the normal artery distally and proximally to the stent *in situ*. On the contrary, Liang et al. (13) deemed to deploy the PED within the indwelling stent, that is to say, placing the proximal and distal ends of the FD device within the previous stent so that the PED would not be stuck at the struts of the previous stent. This resembled the FRED flow diverter, which has the additional benefit of lowering postprocedural ischemia (15). The position of the previous stent is already fixed, and retreatment should be conducted, taking into account the primary treatment and prioritizing individualized treatment. The core of PED deployment is adequate expansion and favorable wall apposition. In our series, we found that deploying the PED only to cover the distal or proximal end of the previous stent was effective in some cases (Figures 2, 5). Optical coherence tomography (OCT) is a diagnostic tool that enables the assessment of stent-strut apposition on the vessel wall and the degree of neointimal endothelialization across the neck of the aneurysm where a previous stent was placed (16, 17). This information can be valuable in guiding the retreatment of aneurysms that have been treated with stents.

To achieve adequate device apposition to the vessel wall, balloon angioplasty may be performed (5). Balloon angioplasty can aid in achieving a complete opening of the PED and ensure that the microwire passes through the central axis of the previous stent, rather than following an “in-out-in.” Additionally, rotating and pushing a J-shaped-tip microwire has proven to be an effective technique when advancing a microwire through a previous stent, preventing the microwire from going through the cells of previous stents effectively (14). Large-diameter intermediate catheters can also be used to test whether the microwire has gone “in-out-in” (6). The application scenarios of this scheme may be limited. In certain scenarios, such as cases involving the post-ICA communication segment or patients with severe atherosclerosis and tortuous blood vessels, passing a large intermediate catheter becomes particularly challenging.

Despite its independent capabilities, many operators continue to use coils during PED deployment. The PED + coils approach was associated with a higher rate of ischemic stroke (1.9% vs. 0.5%) but higher occlusion rates and lower recanalization rates (18). Based on the individual cases mentioned, it seems that the suitability of performing PED + coiling for recurrent aneurysms varies depending on certain factors. For cases where the previously implanted stent was a low porosity stent such as LVIS and the diameter of the parent artery was relatively small, it might be challenging for the microcatheter to penetrate the stent mesh. In addition, if the residual tumor neck/body is small and difficult to fill with coiling, then PED alone may be sufficient. In the mentioned case of patient #3, where an LVIS stent was used in the past and the residual aneurysm was small, PED alone resulted in a complete closure of the aneurysm after 12 months. Contrastingly, when dealing with recurrent aneurysms that have a large neck and high flow rate, performing PED + coiling would be more appropriate. In the case of patient #2, who died of SAH after the operation, highlights the importance of using PED in combination with coiling. In some cases, dense packing of the aneurysm may not be necessary. The flow diversion function of PED and the stagnation function of loose packing can significantly reduce the risk of rebleeding in recurrent aneurysms. Additionally, even if the residual aneurysm does not completely close, the purpose of allowing patients to survive with aneurysms may still be achieved.

There is no consensus on the antiplatelet strategy for PED implantation in the treatment of intracranial aneurysms (19, 20). At our center, patients are placed on daily dual antiplatelet therapy (aspirin, 100 mg/day, and clopidogrel, 75 mg/day, for at least 5 days) prior to PED placement. Tirofiban is administered as an intra-arterial bolus during or immediately after FD deployment (5 mcg/kg), followed by a maintenance dose if necessary (0.08 mcg/kg/min IV infusion, 24–48 h). Postprocedural dual antiplatelet therapy included clopidogrel, 75 mg/day for 6 months, and aspirin, 100 mg/day for 12 months. However, the antiplatelet strategy may be adjusted based on follow-up DSA results and the healing progress of the aneurysm.

For example, we conducted a follow-up of patient #4 in the 4th month after PED implantation, during which the presence of residual contrast agent in the aneurysmal neck was found to be more apparent compared to immediately after the implantation (Figures 5D, I). As a result, the decision was made to discontinue clopidogrel and instead continue with aspirin only. Subsequently, a 10-month follow-up DSA after PED implantation demonstrated a gradual healing of the aneurysm neck (Figure 5L). Accordingly, antiplatelet therapy ceased to promote intra-aneurysm thrombosis. Furthermore, the 16-month follow-up DSA showed further improvement in the healing of the aneurysm neck (Figure 5O).

In cases where in-stent stenosis occurred after PED implantation, it was common practice to prolong dual antiplatelet therapy, in line with previous reports (16). Nevertheless, we largely believed that routine platelet-function testing, as currently conducted, might be unnecessary. This was consistent with the DELPHI consensus statement (21). Furthermore, a recent study revealed that platelet testing was associated with worse clinical outcomes for patients treated with PED (22).

The summary of the studies related to flow diversion deployed in recanalized aneurysms is shown in Table 3.

**Table 3:** Studies show retreatment with a flow diverter for recanalized intracranial aneurysms after stent-assisted coiling.

**References**	**Number of patients**	**Follow-up period (months)**	**Complete closure rate (%)**	**Time interval between initial treatment and FD implantation (months)**
Yan et al. (14)	13	6–36	100%	0.47–3
Li et al. (20)	12	NR	100%	NR
Akgul et al. (7)	18	NR	77.8%	NR
Neira et al. (16)	1	24	100%	12
Park et al. (12)	16	22	94.10%	0.5–16
Bender et al. (18)	9	10.4	57.1%	45.2–155.2
Liang et al. (13)	1	6	100%	9
Topcuoglu et al. (8)	2	NR	100%	NR
Mascitelli et al. (6)	1	6	100%	6
Daou et al. (11)	21	15.67	55.6%	NR
Benaissa et al. (9)	6	NR	NR	NR
Chalouhi et al. (5)	6	NR	NR	NR
Current study	4	6–16	50%	6–13

## Conclusion

The utilization of PED for retreatment in cases of recurrent aneurysms after stent-assisted coiling is a multifaceted procedure demanding meticulous evaluation of individual cases and the implementation of suitable techniques to attain the best possible outcomes. This study also has some major limitations: limited case numbers, retrospective nature, insufficient follow-up time, and single-center nature. These limitations should be considered when interpreting the findings of this study, and future research with larger sample sizes, longer follow-up periods, and appropriate control groups is warranted to further investigate the efficacy and safety of PED deployment in recurrent stented aneurysms.

## Data availability statement

The original contributions presented in the study are included in the article/supplementary material, further inquiries can be directed to the corresponding author.

## Ethics statement

The studies involving humans were approved by the Institutional Research Ethics Boards of Guangdong Sanjiu Brain Hospital. The studies were conducted in accordance with the local legislation and institutional requirements. Written informed consent for participation was not required from the participants or the participants' legal guardians/next of kin in accordance with the national legislation and institutional requirements. Written informed consent was obtained from the individuals for the publication of any potentially identifiable images or data included in this article.

## Author contributions

JL: Methodology, Validation, Visualization, Writing—original draft. YL: Funding acquisition, Methodology, Validation, Visualization, Writing—original draft. LZ: Methodology, Validation, Visualization, Writing—review & editing. XX: Methodology, Validation, Visualization, Writing—review & editing. PW: Supervision, Writing—original draft.
